# A revised circumscription for the Blakeeae (Melastomataceae) with associated nomenclatural adjustments

**DOI:** 10.3897/phytokeys.20.4344

**Published:** 2013-02-22

**Authors:** Darin S. Penneys, Walter S. Judd

**Affiliations:** 1 Department of Botany, California Academy of Sciences, San Francisco, California 94118–4503, U.S.A.; 2 Department of Biology, University of Florida, Gainesville, Florida 32611, U.S.A.

**Keywords:** Blakeeae, *Blakea*, *Chalybea*, *Huilaea*, Melastomataceae, Neotropics, nomenclature, *Topobea*

## Abstract

Systematic investigations and phylogenetic analyses of the Blakeeae (Melastomataceae) have indicated that *Topobea* should be synonymized under *Blakea*, and *Huilaea* under *Chalybea*. Presented here is a detailed description of the Blakeeae, a key to its two accepted genera, and a listing of 62 new combinations, including 3 new names, necessitated by the transfer of *Topobea* as follows: *Blakea acuminata* (Wurdack) Penneys & Judd, **comb. nov.**, *Blakea adscendens* (E.Cotton & Matezki) Penneys & Judd, **comb. nov.**, *Blakea albertiae* (Wurdack) Penneys & Almeda, **comb. nov.**, *Blakea amplifolia* (Almeda) Penneys & Almeda, **comb. nov.**, *Blakea arboricola* (Almeda) Penneys & Almeda, **comb. nov.**, *Blakea asplundii* (Wurdack) Penneys & Judd, **comb. nov.**, *Blakea barbata* (Gleason) Penneys & Judd, **comb. nov.**, *Blakea brenesii* (Standl.) Penneys & Almeda, **comb. nov.**, *Blakea brevibractea* (Gleason) Penneys & Judd, **comb. nov.**, *Blakea bullata* (E.Cotton & Matezki) Penneys & Judd, **comb. nov.**, *Blakea calcarata* (L.Uribe) Penneys & Judd, **comb. nov.**, *Blakea calophylla* (Almeda) Penneys & Almeda, **comb. nov.**, *Blakea calycularis* (Naudin) Penneys & Almeda, **comb. nov.**, *Blakea castanedae* (Wurdack) Penneys & Judd, **comb. nov.**, *Blakea clavata* (Triana) Penneys & Judd, **nom. nov.**, *Blakea cordata* (Gleason) Penneys & Almeda, **comb. nov.**, *Blakea cuprina* Penneys & Judd, **nom. nov.**, *Blakea cutucuensis* (Wurdack) Penneys & Judd, **comb. nov.**, *Blakea dimorphophylla* (Almeda) Penneys & Almeda, **comb. nov.**, *Blakea discolor* (Hochr.) Penneys & Judd, **comb. nov.**, *Blakea dodsonorum* (Wurdack) Penneys & Almeda, **comb. nov.**, *Blakea eplingii* (Wurdack) Penneys & Judd, **comb. nov.**, *Blakea ferruginea* (Gleason) Penneys & Judd, **comb. nov.**, *Blakea fragrantissima* (Almeda) Penneys & Almeda, **comb. nov.**, *Blakea gerardoana* (Almeda) Penneys & Almeda, **comb. nov.**, *Blakea glaberrima* (Triana) Penneys & Judd, **comb. nov.**, *Blakea henripittieri* (Cogn.) Penneys & Almeda, **comb.** et **nom. nov.**, *Blakea hexandra* (Almeda) Penneys & Almeda, **comb. nov.**, *Blakea horologica* Penneys & Judd, **nom. nov.**, *Blakea induta* (Markgr.) Penneys & Judd, **comb. nov.**, *Blakea inflata* (Triana) Penneys & Judd, **comb. nov.**, *Blakea insignis* (Triana) Penneys & Judd, **comb. nov.**, *Blakea intricata* (Almeda) Penneys & Almeda, **comb. nov.**, *Blakea killipii* (Wurdack) Penneys & Judd, **comb. nov.**, *Blakea lentii* (Almeda) Penneys & Almeda, **comb. nov.**, *Blakea longiloba* (Wurdack) Penneys & Judd, **comb. nov.**, *Blakea longisepala* (Gleason) Penneys & Judd, **comb. nov.**, *Blakea macbrydei* (Wurdack) Penneys & Judd, **comb. nov.**, *Blakea maguirei* (Wurdack) Penneys & Judd, **comb. nov.**, *Blakea maurofernandeziana* (Cogn.) Penneys & Almeda, **comb. nov.**, *Blakea mcphersonii* (Almeda) Penneys & Almeda, **comb. nov.**, *Blakea modica* (Wurdack) Penneys & Judd, **comb. nov.**, *Blakea mortoniana* (Wurdack) Penneys & Judd, **comb. nov.**, *Blakea muricata* (Lozano) Penneys & Judd, **comb. nov.**, *Blakea pascoensis* (Wurdack) Penneys & Judd, **comb. nov.**, *Blakea pluvialis* (Standl.) Penneys & Almeda, **comb. nov.**, *Blakea sessilifolia* (Triana) Penneys & Judd, **comb. nov.**, *Blakea setosa* (Triana) Penneys & Judd, **comb. nov.**, *Blakea standleyi* (L.O.Williams) Penneys & Almeda, **comb. nov.**, *Blakea stephanochaeta* (Naudin) Penneys & Judd, **comb. nov.**, *Blakea steyermarkii* (Wurdack) Penneys & Judd, **comb. nov.**, *Blakea suaveolens* (Almeda) Penneys & Almeda, **comb. nov.**, *Blakea subbarbata* (Wurdack) Penneys & Judd, **comb. nov.**, *Blakea subscabrula* (Triana) Penneys & Judd, **comb. nov.**, *Blakea subsessiliflora* (Wurdack) Penneys & Judd, **comb. nov.**, *Blakea superba* (Naudin) Penneys & Judd, **comb. nov.**, *Blakea tetramera* (Almeda) Penneys & Almeda, **comb. nov.**, *Blakea tetroici* (Wurdack) Penneys & Judd, **comb. nov.**, *Blakea toachiensis* (Wurdack) Penneys & Judd, **comb. nov.**, *Blakea trianae* (Cogn.) Penneys & Judd, **comb. nov.**, *Blakea verrucosa* (Wurdack) Penneys & Judd, **comb. nov.**, *Blakea watsonii* (Cogn.) Penneys & Almeda, **comb. nov.**

## Introduction

Melastomataceae Juss., with approximately 5000 species and 190 genera (Stevens 2001–), is one of the ten largest families of angiosperms. The tribe Blakeeae Bentham & Hooker is strictly Neotropical, with centers of diversity in the “megadiverse” Choco-Andean region of South America and the mountains of Costa Rica and Panama, though the species range from Chiapas, Mexico, to the Amazon of Bolivia and Brazil, to French Guiana. Three additional species are found in the West Indies. Members of this tribe are notable for their often large, showy flowers that attract a diversity of pollinators including bees, birds, bats, and rodents (Lumer 1980). Mites and ants live in mutualistic associations in leaf and stem domatia of many Blakeeae (Penneys and Judd 2011). Numerous species in this tribe have great horticultural potential, are relatively easy to grow in temperate zone greenhouses, but are rarely cultivated.

The Blakeeae, as historically circumscribed, comprises nearly 200 species in two genera, *Blakea* L. and *Topobea* Aubl. However, morphological, molecular, and combined phylogenetic analyses (Penneys et al. 2004; Penneys 2007; Penneys and Judd 2010, 2011, in press) have necessitated adjustments in the circumscription of the Blakeeae. Morphological characters pertaining to the androecium that have been used as a basis for separating these two genera have proven to be homoplasious and of limited taxonomic value (Penneys 2007, Penneys and Judd 2011, Penneys and Judd in press). Recognition of *Topobea* renders both that genus and *Blakea* polyphyletic, thus *Topobea* is here relegated to synonymy under *Blakea*. According to Cogniaux (1891), the filaments in *Blakea* are thick, while in *Topobea* they are filiform. The latter character state was found in only three species of *Topobea*, and even the generotype, *Topobea parasitica* Aubl., was not one of them. The anthers of *Blakea* have been said (Cogniaux 1891, Almeda 2000a) to be laterally compressed, while those of *Topobea* are rounded. Close examination of *Topobea* anthers proves that they are also laterally flattened, though since the anthers are generally more subulate, this fact is less apparent than in *Blakea* (Penneys 2007, Penneys and Judd 2011). Morphological characters synapomorphic for an expanded *Blakea* include the prevalent but not exclusively hemiepiphytic habit; axillary, nonramified truncate monotelic synflorescences, flowers subtended by two pairs of decussate bracts, external calyx teeth lacking, flowers zygomorphic due to the declinate androecium, and anthers laterally compressed (Penneys and Judd 2011).

*Chalybea* Naudin and *Huilaea* Wurdack, were formerly placed in the Miconieae (Naudin 1852, Triana 1871, Wurdack 1957, Judd and Skean 1991), presumably on the basis of their having berry fruits. Morphological and molecular phylogenetic analyses demonstrate that the two genera properly belong in the Blakeeae, forming a clade with ten species sister to *Blakea* (Penneys et al. 2004, Penneys 2007, Penneys and Judd 2010, Penneys and Judd 2011, Penneys and Judd in press, Morales-P 2010, Morales-P and Penneys 2010, Morales-P et al. submitted). *Chalybea* has been found to be nested within *Huilaea* (Morales-P 2010, Morales-P and Penneys 2010, Penneys and Judd 2010, Morales-P et al. submitted), thus the species in the latter genus will be transferred (Morales-P 2010, Morales-P et al. in prep) to *Chalybea*, which has nomenclatural priority. The inclusion of *Chalybea* in the Blakeeae necessitates an expansion of the recognized morphological variation within the tribe (see below). The expanded *Chalybea* has numerous morphological synapomorphies including the terrestrial, arborescent habit, pinwheel acarodomatia in the vein axils, truncate monotelic synflorescences with elongate peduncles (Mora-Osejo 1966), actinomorphic, pseudocampanulate flowers subtended by a single pair of narrow bracteoles, lenticellate hypanthia, anthers laterally rounded and relatively short compared to the filament length, inferior ovaries, styles not immersed in a crown, and yellowish-green fruits with thick and leathery exocarps.

In this paper, we present a revised description of the Blakeeae, a key to *Blakea* and *Chalybea* with diagnoses of each, and an enumeration of the new names and combinations necessitated by the transfer of *Topobea* to *Blakea*.

## Taxonomy and nomenclature

### Blakeeae

Benth. & Hook. f., Gen. Pl. 1: 727, 735. 1867.

Pyxidantheae Triana, Bull. Congr. Int. Bot. Amsterdam. 457. 1865. Type genus: *Pyxidanthus* Naudin.

#### Type genus:

*Blakea* P.Browne.

#### Remarks.

Evergreen shrubs, trees, or lianas, growing as terrestrials, hemiepiphytes, or epiphytes, with variable indumentum, the hairs sparsely to densely distributed, unicellular or multicellular, variously smooth to roughened to barbellate, furfuraceous-granulose, eglandular, or sessile to short- to long-stalked globular glandular, setae slender to stoutly conic, occasionally apically fimbriate. Twigs rectangular, square, quadrate, to terete in cross-section, sometimes formicarial with hollow or apically inflated internodes and subnodal entrance holes. Stipules absent, interpetiolar and ± coriaceous, or layered and membranaceous. Petioles terete, canaliculate, to winged; leaves opposite, decussate, nearly sessile to petiolate, equal to anisophyllous, then the smaller leaf sometimes deciduous; blade chartaceous to coriaceous, flat to verrucose, frequently drooping and vivid yellow to scarlet when senescent, the apex acute, to broadly rounded, often abruptly short to long acuminate, the base acute, to rounded, to cordate, rarely subpeltate or decurrent along petiole margins, the margin plane to revolute, entire to toothed; venation acrodromous, basal to plinerved, with prominent midvein and 2 to 7 pairs of secondary veins (including a pair of weak, submarginal veins), tertiary veins numerous and striolate to widely spaced, subperpendicular to midvein; adaxial surface usually glabrescent, but sometimes with persistent hairs (as above), the veins variously flat to impressed; abaxial surface light to dark green or tan, essentially glabrous to densely pubescent with various hair types (as above), the midvein and major secondary veins raised, minor secondary veins, tertiary veins and higher order veins flat to raised; acarodomatia frequently present in primary-axillary vein axils, formed by hair tufts, coalesced veins, or membranes, or rarely a foliose flap of tissue partially encircling the adaxial apex of the petiole (*Blakea austin-smithii*, *Blakea chlorantha*). Inflorescences axillary in distal nodes, simple or compound cyme (*Chalybea*) or solitary to fasciculate (*Blakea*), bracts and bracteoles caducous (*Chalybea*) or persistent (*Blakea*), each flower subtended by a single pair (*Chalybea*) or two (very rarely three) pairs (*Blakea*) of bracteoles, the bracteoles obscure to foliaceous, membranaceous to coriaceous, free to completely connate, appressed to hypanthium or spreading, linear to elliptic to oblate, entire to remotely denticulate, with pubescence as above. Flowers perfect, 6–merous (4–merous in *Blakea tetramera*), mostly showy, actinomorphic to zygomorphic as a result of the declinate androecium (*Blakea*), frequently with pleasantly sweet to musky fragrance (*Blakea*), rarely nectariferous, the stomatal nectaries located on the anther connective appendages. Hypanthium narrowly to broadly globose, cylindrical to conical, terete to costate, the outer surface glabrous or with pubescence (as above), when present, hairs usually denser proximally, the inner surface glabrous or rarely glandular-pubescent, obscurely to prominently ridged, the apices of the ridges not to distinctly projecting around style base. External calyx teeth, when present, (4–) 6 (absent in calyptrate species), distinct, with apex acuminate to acute, or reduced to blunt thickenings; internal calyx lobes (4–) 6 (absent in calyptrate species), valvate or rarely imbricate, the lobes merely inconspicuous tubercles, to narrowly to broadly triangular, truncate, lanceolate, to orbicular, rarely with a large flap of tissue elaborated from the apical and distal portion of the calyx lobe, then tightly held to the underside of the lobe (*Blakea bocatorena* ined., *Blakea calycosa*, *Blakea tuberculata*), the margin entire, often callose-thickened, in fruit, the lobes sometimes becoming colorful, sometimes inrolled; calyx tube glabrous inside. Petals (4–) 6, rarely containing druse crystals, imbricate in bud, orbiculate, ovate, elliptic, obovate, to rhombic, frequently widely so, sometimes clawed, symmetrical or oblique, reflexed, rotate, or pseudocampanulate, white, cream, pink, lavender, magenta, red, or green, the apex acute, obtuse, rounded, truncate, to emarginate; margin entire to minutely erose; both surfaces usually glabrous, rarely sparsely pubescent. Stamens 12 (6 and antesepalous in the hexandrous *Blakea* clade; 8 in *Blakea tetramera*), incurved in bud, isomorphic or rarely subequal with central stamens slightly larger than those at perimeter of cycle (*Blakea*); filaments in cross section nearly flat dorsally, usually with an obscure to prominent ventral keel and laterally narrowed (rarely cylindrical), white, cream, pink, or lavender; anthers white, cream, yellow, bluish, lavender, to deep purple, free or connate, laterally rounded to flattened, anther sacs somewhat to deeply separated ventrally, linear to obtuse, opening by one or two pores, the pores sometimes confluent, dorsally to ventrally positioned; dorsal basal anther connective appendages smooth to rugose, mostly modified blunt knobs, parallel longitudinal ridges, triangular spurs (sometimes two present), or caudate. Ovary (2–) 4–6 (–12)–loculate, inferior to superior, apically glabrous or rarely glandular-pubescent, smooth to ridged, unadorned or with circumstylar, short- to long-acute projections, ± rectangular flanges, or rarely with ascending, radiating, elongate appendages (*Blakea glandulosa*, *Blakea hirsuta*); placentation axile to deeply intruded axile, the ovules numerous, anatropous; style elongate (bluntly clavate in *Blakea princeps*), terete, cylindrical, slightly swollen suprabasally, or tapered, glabrous or glandular-pubescent, white, cream, pink, or lavender; stigma truncate to capitate, rarely obscurely lobed and concave (mostly in *Chalybea*). Berries ± globose to elliptical, greenish when immature, becoming yellowish-green, pale greenish-white, red, lavender, orange, or deep purple at maturity, glabrous to pubescent (as above), the exocarp thin to leathery, fairly dry and unpalatable to juicy, sweet, and highly comestible (especially *Chalybea*). Seeds numerous, pyramidal to ovoid, testa smooth to sculpted.

Distribution: Mexico (Chiapas) to Bolivia and Brazil; Jamaica, Lesser Antilles. The Pacific slopes of the Cordillera Occidental, Colombia represent the center of specific and morphological diversity. Occurring from sea level to ca. 3000 meters.

#### Key to the genera of Blakeeae:

**Table:** 

1a	Flowers solitary or fasciculate, each flower subtended by two (three) pairs of expanded (rarely lanceolate), persistent, decussate bracts	*Blakea*
1b	Flowers in simple or compound cymes, each flower subtended by a single pair of lanceolate, cauducous bracts	*Chalybea*

### Chalybea

Naudin, Ann. Sci. Nat., Bot. sér. 3 16: 99. 1851.

http://species-id.net/wiki/Chalybea

Huilaea Wurdack, Brittonia 9: 106. 1957. Type: *Huilaea penduliflora* Wurdack, Brittonia 9: 106. 1957. **TYPE. COLOMBIA.Huila:** Cordillera Oriental, wet temperate forest of deep moist canyon near camp on north side of Río Venadito, 25 km SE of La Bodega, 2450 m, 1 Dec 1944, E.Little 9101 (holotype: US!; isotypes: COL!, NY!).

#### Type.

*Chalybea corymbifera* Naudin, Ann. Sci. Nat., Bot. sér. 3 16: 100. 1851. TYPE. COLOMBIA. Santander: Pamplona, La Baja, ca. 3000 m, Dec 1846, Funck & Schlim 1312 (holotype: P!, photos at NY!, US!; isotypes: BM!, BR! (2 sheets), G!, photos of G sheet at NY!, US!).

#### Remarks.

**Phylogenetic analyses** (Morales-P 2010, Morales-P et al. submitted, Penneys and Judd 2010) indicate that *Chalybea* and *Huilaea* form a strongly-supported clade. However, as *Chalybea* is nested within *Huilaea*, species belonging to the latter genus must be transferred because *Chalybea* has nomenclatural priority. *Chalybea* has been distinguished from *Huilaea* by having inflorescences with 21–39 flowers (vs. 3–17 in *Huilaea*), the flowers 13–20 mm long (vs. 45–55 mm long), the hypanthium 7–10 mm long and 8–10 mm wide (vs. 13–31 mm long x 11–19 mm wide), the petals 12–14 mm long and 5–7 mm wide (vs. 26–44 mm long x 10–22 mm wide), and white to cream or green, sometimes pink–tinged apically (vs. pink to red, paler inside), the anthers 3–4 mm long (vs. 7–10 mm long), and the style 10–12 mm long (vs. 22–33 mm long).

*Chalybea* includes ten described species of small, South American trees: seven endemic to Colombia, two to Ecuador, and one to Peru. All are found in the Andes, except one species that is restricted to the Sierra Nevada de Santa Marta, Colombia.

For more information on the systematics of *Chalybea* (and *Huilaea*), see Lozano-Contreras and Ruiz-R (1996), Morales-P and González (2005), Morales-P (2010), Morales-P and Penneys (2010), and Morales-P et al. (submitted).

### Blakea

P.Browne, Civ. Nat. Hist. Jamaica 323. tab. 35. 1756.

http://species-id.net/wiki/Blakea

Topobea Aubl., Hist. Pl. Guiane 1: 476. 1775. Type: *Topobea parasitica* Aubl., Hist. Pl. Guiane 1: 476. 1775. TYPE. FRENCH GUIANA. Aublet s.n. (holotype: BM!).
Valdesia Ruiz & Pav., Fl. Peruv. Prodr. 67. 1794. Type: *Valdesia repens* Ruiz & Pav., Syst. Veg. Fl. Peruv. Chil. 121. 1798. TYPE. PERU. Ruiz & Pav. s.n. (holotype: B (destroyed), photos at MO!, US!).
Pyxidanthus Naudin, Ann. Sci. Nat. Bot. ser 3 18: 150. 1852. Type: *Pyxidanthus schlimii* Naudin, Ann. Sci. Nat., Bot. sér. 3 18: 151. 1852. TYPE. VENEZUELA. Trujillo, Funck & Schlim 738 (holotype: G; (fragment), photos at MO!, US!; isotypes: BM!, BR!, MPU!).
Amaraboya Linden, Illustr. Hort. 34: 15. 1887. Type: *Amaraboya princeps* Linden, Illustr. Hort. 34: 15. 1887. (lectotype, designated here: *pl*. IV of the protologue).

#### Type.

*Blakea trinervia* L., Syst. Nat. ed. 10: 1044. 1759. TYPE. JAMAICA. P.Browne s.n. (holotype: LINN-612.1!).

#### Remarks.

*Blakea* is characterized by solitary or fasciculate axillary inflorescences, and flowers subtended by two (rarely three) pairs of decussate, (usually) expanded, subtending bracts. Additionally, *Blakea* may usually be distinguished from *Chalybea* by the species often being hemiepiphytic or epiphytic, mostly with rotate corollas, anthers that are usually laterally flattened and often connate and/or deflexed, with the connective appendages generally better developed and of diverse morphology, and many *Blakea* species have ovaries that are not entirely inferior, often possessing stylar collars. Cladistic analyses (Penneys et al. 2004, Penneys 2007, Penneys and Judd 2011, in press) have provided conclusive evidence that the recognition *Topobea* renders both that genus and *Blakea* polyphyletic, thus *Topobea* is here relegated to synonymy under *Blakea*.

*Blakea* comprises approximately 180 species distributed from Mexico (Chiapas) to Bolivia and Brazil, with two species in Jamaica and one in the Lesser Antilles.

Formal nomenclatural transfers to *Blakea* are made below for 62 species of *Topobea* that were not treated by earlier workers who made similar new combinations (e.g., Don 1823, Macbride 1941). Three of these include new specific epithets for names already occupied in *Blakea*.

Important references concerning the systematics of *Blakea* include (Almeda 1990, 2000a, 2000b, 2001a, 2001b, 2009, Gleason 1945, Wurdack 1973, 1980, Penneys 2007, Penneys and Judd 2011, in press).

### Nomenclatural changes:

***Blakea acuminata* (Wurdack) Penneys & Judd, comb. nov.** Basionym: *Topobea acuminata* Wurdack, Phytologia 52: 69. 1982. **TYPE. ECUADOR. Pastaza:** in remnants of primary rain forest at Tarqui 5 km south of Puyo, ca. 850 m, 8 Mar 1980, G.Harling & L.Anderson 17058 (holotype: GB; isotype: US!). IPNI ID: urn:lsid:ipni.org:names:77124962-1

***Blakea adscendens* (E.Cotton & Matezki) Penneys & Judd, comb. nov.** Basionym: *Topobea adscendens* E. Cotton & Matezki, Brittonia 55: 76. 2003. **TYPE. ECUADOR. Zamora-Chinchipe:** San Francisco Research Station, ca. 30 km from the city of Loja on hwy. toward Zamora, 2050 m, 03°58'18"S, 79°04'44"W, 2 Dec 2000, S.Matezki 344 (holotype: AAU; isotypes: LOJA!, MO!, NY!, QCNE!, UBT). IPNI ID: urn:lsid:ipni.org:names:77124963-1

***Blakea albertiae* (Wurdack) Penneys & Almeda, comb. nov.** Basionym: *Topobea albertiae* Wurdack, Phytologia 55: 146. 1984. **TYPE. COLOMBIA. Antioquia:** Fincas Montepinar and Las Palmas, Vereda Quebrada Larga, Municipio Guatapé at the line with Municipio San Rafael, 1800 m, 4 Sep 1982, L.Albert de Escobar et al. 2278 (holotype: HUA; isotypes: COL!, JUAM!, US!). IPNI ID: urn:lsid:ipni.org:names:77124964-1

***Blakea amplifolia* (Almeda) Penneys & Almeda, comb. nov.** Basionym: *Topobea amplifolia* Almeda, Proc. Calif. Acad. Sci., ser. 4, 52: 518. 2001. **TYPE. COSTA RICA. Limón:** Cantón de Talamanca, Bratsi, Amubri, Alto Lari, Kivut. Afluente innominado del Río Lari, margen izquierda, 1200 m, 09°23'25"N, 83°04'25"W, 21 Mar 1992, G.Herrera 5407 (holotype: CAS!; isotypes: CR!, INB!, MO!). IPNI ID: urn:lsid:ipni.org:names:77124965-1

***Blakea arboricola* (Almeda) Penneys & Almeda, comb. nov.** Basionym: *Topobea arboricola* Almeda, Proc. Calif. Acad. Sci., ser. 4, 52: 98. 2000. **TYPE. PANAMA. Bocas del Toro/Chiriquí border:** windswept cloud forest on slopes and valleys of the Cerro Colorado region, 1450 m, 27 Jan 1989, F.Almeda et al. 6456 (holotype: CAS!; isotypes: MO!, PMA!). IPNI ID: urn:lsid:ipni.org:names:77124966-1

***Blakea asplundii* (Wurdack) Penneys & Judd, comb. nov.** Basionym: *Topobea asplundii* Wurdack, Phytologia, 29: 149. 1974. **TYPE. ECUADOR. Napo:** collected between Tena and Napo, 5 Jan 1940, E.Asplund 10254 (holotype: S; isotypes: BR!, LL!, NY!). IPNI ID: urn:lsid:ipni.org:names:77124967-1

***Blakea barbata* (Gleason) Penneys & Judd, comb. nov.** Basionym: *Topobea barbata* Gleason, Bull. Torrey Bot. Club 72: 393. 1945. **TYPE. COLOMBIA. Valle del Cauca:** collected at Barco, on the Pacific coast, Río Cajambre, 5–80 m, J.Cuatrecasas 17215 (holotype: NY!; isotypes: COL!, F!). IPNI ID: urn:lsid:ipni.org:names:77124968-1

***Blakea brenesii* (Standl.) Penneys & Almeda, comb. nov.** Basionym: *Topobea brenesii* Standl., Publ. Field Mus. Nat. Hist., Bot. Ser. 18: 842. 1938. **TYPE. COSTA RICA. Alajuela:** La Palma de San Ramón, 1250 m, Mar 1929, A.Brenes 6732 (holotype: F!; isotypes: CR, NY!). IPNI ID: urn:lsid:ipni.org:names:77124969-1

***Blakea brevibractea* (Gleason) Penneys & Judd, comb. nov.** Basionym: *Topobea brevibractea* Gleason, Brittonia 2: 326. 1937. **TYPE. ECUADOR. Cotopaxi [as León]:** collected near Hacienda Solento, Santa Rosa, Canton Pajili, 1000 m, Y.Mexia 6683 (holotype: NY!; isotypes: CAS!, F!, K!, US!). IPNI ID: urn:lsid:ipni.org:names:77124970-1

***Blakea bullata* (E.Cotton & Matezki) Penneys & Judd, comb. nov.** Basionym: *Topobea bullata* E. Cotton & Matezki, Brittonia 55: 78. 2003. **TYPE. ECUADOR. Zamora-Chinchipe:** San Francisco Biological Station, ca. 30 km from the city of Loja on hwy. toward Zamora, 2100 m, 03°58'18"S, 79°04'44"W, 5 Sept 2001, S.Matezki 396 (holotype: AAU; isotypes: LOJA!, MO!, NY!, QCNE!, UBT). IPNI ID: urn:lsid:ipni.org:names:77124971-1

***Blakea calcarata* (L.Uribe) Penneys & Judd, comb. nov.** Basionym: *Topobea calcarata* L.Uribe, Caldasia 11: 89. 1971. **TYPE. COLOMBIA. Chocó:** Arusi, 17 Feb 1947, O.Haught 5579 (holotype: COL!; isotypes: NY!, US!). IPNI ID: urn:lsid:ipni.org:names:77124972-1

***Blakea calophylla* (Almeda) Penneys & Almeda, comb. nov.** Basionym: *Topobea calophylla* Almeda, Proc. Calif. Acad. Sci 43: 281. 1984. **TYPE. PANAMA. Veraguas:** 5 mi. W of Santa Fé on road past Escuela Agricola Alto Piedra on Pacific side of divide, 800–1200 m, 18 Mar 1973, T.Croat 23000 (holotype: CAS!; isotypes: MEXU, MO!, PMA!, US!). IPNI ID: urn:lsid:ipni.org:names:77124973-1

***Blakea calycularis* (Naudin) Penneys & Almeda, comb. nov.** Basionym: *Topobea calycularis* Naudin, Ann. Sci. Nat., Bot., sér. 3,: 149. 1852. **TYPE. MEXICO. Chiapas.** Zuluzuchiapas, April, J.Linden 650 (holotype: P!; isotypes: BR!, K!). IPNI ID: urn:lsid:ipni.org:names:77124975-1

***Blakea castanedae* (Wurdack) Penneys & Judd, comb. nov.** Basionym: *Topobea castanedae* Wurdack, Phytologia 7: 244. 1960. **TYPE. COLOMBIA. Nariño:** La Guayacana, Tumaco, 27 June 1951, R.Castañeda 2939 (holotype: NY!; isotype: F!). IPNI ID: urn:lsid:ipni.org:names:77124974-1

***Blakeaclavata* (Triana) Penneys & Judd, nom. nov.** Basionym: *Topobea gracilis* Triana, Trans. Linn. Soc. 28: 150. 1871 [1872]. **TYPE. COLOMBIA. Nariño: **Barbacoas, 400 m, 1854–1857, J.Triana 4085b (holotype: BM!).The specific epithet is preempted by *Blakea gracilis* Hemsl., Diag. Pl. Nov. 13. 1878. The new specific epithet is from the Latin *clavata*, meaning club, in reference to the club-shaped, formicarial internodes. IPNI ID: urn:lsid:ipni.org:names:77124977-1

***Blakea cordata* (Gleason) Penneys & Almeda, comb. nov.** Basionym: *Topobea cordata* Gleason, Phytologia 3: 354. 1950. **TYPE. PANAMA. Coclé:** Cerro Pajita, hills north of El Valle de Antón, 1000–1200 m, 7 Feb 1947, P.Allen & D.Allen 4178 (holotype: NY!; isotype: MO!). IPNI ID: urn:lsid:ipni.org:names:77124978-1

***Blakeacuprina* Penneys & Judd, nom. nov.** Basionym: *Topobea glabrescens* Triana, Trans. Linn. Soc. 28: 149. 1871 [1872]. **TYPE. COLOMBIA. Nariño:** Barbacoas, 900 m, May 1853, J.Triana 4100 (holotype: BM!; isotype: COL!).The specific epithet is preempted by *Blakea glabrescens* Benth., Bot. Voy. Sulph. 94. 1844. The new specific epithet for this species is derived from the Latin *cuprina*, meaning copper, in reference to the dense, coppery pubescence on the abaxial leaf surfaces. IPNI ID: urn:lsid:ipni.org:names:77124979-1

***Blakea cutucuensis* (Wurdack) Penneys & Judd, comb. nov.** Basionym: *Topobea cutucuensis* Wurdack, Mem. New York Bot. Gard. 16: 45. 1967. **TYPE. ECUADOR. Morona-Santiago/Zamora-Chinchipe:** ridge ascending into central Cutucú, 770 m, 17 Nov–5 Dec 1944, W.Camp E-1129 (holotype: US!; isotype: NY!). IPNI ID: urn:lsid:ipni.org:names:77124980-1

***Blakea dimorphophylla* (Almeda) Penneys & Almeda, comb. nov.** Basionym: *Topobea dimorphophylla* Almeda, Proc. Calif. Acad. Sci., ser. 4, 52: 523. 2001. **TYPE. COSTA RICA. Heredia:** along Río Peje about 0.5 km SW of back end of Vargas property; approximately in the area where an imaginary line drawn between Magsasay (colonia penal) and Puerto Viejo de Sarapiquí would cross the Río Peje, 20 Feb 1982, B.Hammel 11217 (holotype: CAS!; isotypes: CR, DUKE, F!, INB!, MO!, NY!, US!). IPNI ID: urn:lsid:ipni.org:names:77124981-1

***Blakea discolor* (Hochr.) Penneys & Judd, comb. nov.** Basionym: *Topobea discolor* Hochr., Bull. New York Bot. Gard 6: 282. 1910. **TYPE. COLOMBIA. Antioquia:** Truando, 1857, Schott XII (holotype: NY!). IPNI ID: urn:lsid:ipni.org:names:77124982-1

***Blakea dodsonorum* (Wurdack) Penneys & Almeda, comb. nov.** Basionym: *Topobea dodsonorum* Wurdack, Phytologia 38: 304. 1978. **TYPE. ECUADOR. Pichincha-Los Rios border:** in cloud forest along ridge line near La Centinella at Km 12 on road from Patricia Pilar to Flor de Mayo, Montaña de Ila, 600 m, 16 July–11 Aug 1977, C.Dodson & H.Dodson 6752 (holotype: US!; isotypes: MO!, SEL!). IPNI ID: urn:lsid:ipni.org:names:77124983-1

***Blakea eplingii* (Wurdack) Penneys & Judd, comb. nov.** Basionym: *Topobea eplingii* Wurdack, Phytologia 29: 151. 1974. **TYPE. ECUADOR. Esmeraldas:** primary rain forest at Tobar Donoso, junction of Río San Juan and Río Camumbi, 150 m, 01°10'N, 78°31'W, 25 July 1966, C.Jativa & C.Epling 1123 (holotype: US!; isotypes: NY!, US!). IPNI ID: urn:lsid:ipni.org:names:77124984-1

***Blakea ferruginea* (Gleason) Penneys & Judd, comb. nov.** Basionym: *Topobea ferruginea* Gleason, Bull. Torrey Bot. Club 58: 434. 1931. **TYPE. VENEZUELA. Amazonas:** Camp Woods, Savanna Hills, Summit of Mount Duida, 1350 m, Aug 1928–Apr 1929, G.Tate 850 (holotype: NY!; isotype: US!). IPNI ID: urn:lsid:ipni.org:names:77124985-1

***Blakea fragrantissima* (Almeda) Penneys & Almeda, comb. nov.** Basionym: *Topobea fragrantissima* Almeda, Proc. Calif. Acad. Sci 46: 318. 1990. **TYPE. PANAMA. Chiriquí:** vicinity of Fortuna Dam, along trail across valley of Río Hornito, 1100–1250 m, 12 Mar 1988, F.Almeda et al. 6086 (holotype: CAS!; isotypes: CR, F!, MO!, PMA!, TEX!, US!). IPNI ID: urn:lsid:ipni.org:names:77124986-1

***Blakea gerardoana* (Almeda) Penneys & Almeda, comb. nov.** Basionym: *Topobea gerardoana* Almeda, Proc. Calif. Acad. Sci., ser. 4, 52: 527. 2001. **TYPE. COSTA RICA. Limón:** Cordillera de Talamanca between Quebrada Kuisa and Rio Lari, 2100 m, 09°20'25"N, 83°13'45"W, 17 Mar 1993, G.Herrera 5914 (holotype: CAS!; isotypes: CR, INB!, MO!). IPNI ID: urn:lsid:ipni.org:names:77124987-1

***Blakea glaberrima* (Triana) Penneys & Judd, comb. nov.** Basionym: *Topobea glaberrima* Triana, Trans. Linn. Soc. 28: 150. 1871. **TYPE. COLOMBIA. Chocó:** La Cueva, 1200 m, May 1853, J.Triana 4090 (holotype: BM!; isotypes: COL!, G-DC!; K!, P!). IPNI ID: urn:lsid:ipni.org:names:77124992-1

***Blakea henripittieri* (Cogn.) Penneys & Almeda, comb. et nom. nov.** Basionym: *Topobea pittieri* Cogn., Monogr. Phan 7: 1088. 1891. **TYPE. COSTA RICA.** La Palma, 1550 m, 18 Dec 1888, H.Pittier 706 (holotype: BR!; isotypes: BR!, CR!).

The specific epithet is preempted by *Blakea pittierii* Cogn., Monogr. Phan 7: 1080. 1891, which itself is a synonym of *Blakea grandiflora* Hemsl., Diag. Pl. Nov. Mexic. 1: 13. 1878. IPNI ID: urn:lsid:ipni.org:names:77124988-1

***Blakea hexandra* (Almeda) Penneys & Almeda, comb. nov.** Basionym: *Topobea hex- andra* Almeda, Proc. Calif. Acad. Sci 46: 320. 1990. **TYPE. PANAMA. Panamá:** Cerro Jefe, along summit road and along trail into the Chagres Valley, ca. 900 m, 19 Feb 1988, F.Almeda et al. 5837 (holotype: CAS!; isotypes: CR, DUKE, F!, MO!, NY!, PMA!, TEX!, US!). IPNI ID: urn:lsid:ipni.org:names:77124989-1

***Blakea horologica* Penneys & Judd, nom. nov.** Basionym: *Topobea caudata* Wurdack, Phytologia 48: 251. 1981. TYPE. ECUADOR. Carchi: near El Pailon ca. 45 km below Maldonado along path to Tobar Donoso, 800 m, 26 Nov 1979, M.Madison & L.Besse 6991 (holotype: US!; isotype: SEL!). The specific epithet is preempted by Blakea caudata Triana, Trans. Linn. Soc. 28: 148. 1871 [1872]. The new specific epithet is derived from the Latin horologium, meaning hourglass, in reference to the shape of the mature fruits that are distinctly constricted above the ovary. IPNI ID: urn:lsid:ipni.org:names:77124976-1

***Blakea induta* (Markgr.) Penneys & Judd, comb. nov.** Basionym: *Topobea induta* Markgr., Notizblatt Bot. Gart. Berlin-Dahlem 15: 382. 1941. **TYPE. ECUADOR. Pastaza:** Mera, Rio Tigre, 12 Dec 1938, H.Schultze-Rhonhof 3089 (holotype: B, destroyed). IPNI ID: urn:lsid:ipni.org:names:77124990-1

***Blakea inflata* (Triana) Penneys & Judd, comb. nov.** Basionym: *Topobea inflata* Triana, Trans. Linn. Soc. 28: 150. 1871 [1872]. **TYPE. COLOMBIA. Nariño:** inter Tuquerres et Barbacoas, May 1853, J.Triana 4085 (holotype: BM!; isotype: P!). IPNI ID: urn:lsid:ipni.org:names:77124991-1

***Blakea insignis* (Triana) Penneys & Judd, comb. nov.** Basionym: *Topobea insignis* Triana, Trans. Linn. Soc. 28: 150. 1871 [1872]. **TYPE. COLOMBIA. Nariño:** El Paramo inter Tuquerres et Barbacoas, 1100 m, May 1853, J.Triana 4088 (holotype: BM!). IPNI ID: urn:lsid:ipni.org:names:77124993-1

***Blakea intricata* (Almeda) Penneys & Almeda, comb. nov.** Basionym: *Topobea intricata* Almeda, Brittonia, 53: 157. 2001. **TYPE. COSTA RICA. Cartago:** Hwy. #224 on property of ICE hydroelectric plant (now Tapantí National Park) ca. 20–24 km E of the church in Orosí, 1500–1800 m, 5 Jan 1974, F.Almeda et al. 2366 (holotype: CAS!; isotypes: BM!, CR, DUKE, INB!, MEXU, MO!, NY!). IPNI ID: urn:lsid:ipni.org:names:77124994-1

***Blakea killipii* (Wurdack) Penneys & Judd, comb. nov.** Basionym: *Topobea killipii* Wurdack, Phytologia 6: 7. 1957. **TYPE. COLOMBIA. Valle del Cauca:** Buena-ventura Bay, 13 Apr 1939, E.Killip 34982 (holotype: NY!; isotypes: COL!, US!). IPNI ID: urn:lsid:ipni.org:names:77124995-1

***Blakea lentii* (Almeda) Penneys & Almeda, comb. nov.** Basionym: *Topobea lentii* Almeda, Brittonia 53: 160. 2001. **TYPE. COSTA RICA. Cartago:** 3 km E of Cachí, beside Río Naranjo, 1300 m, 11 Jul 1971, R.Lent 2000 (holotype: MO!; isotypes: BM!, CR, DUKE, F!, G, PMA!, US!). IPNI ID: urn:lsid:ipni.org:names:77124996-1

***Blakea longiloba* (Wurdack) Penneys & Judd, comb. nov.** Basionym: *Topobea longiloba* Wurdack, Phytologia 6: 8. 1957. **TYPE. COLOMBIA. Putumayo:** alta cuenca del río Putumayo en el Valle de Sibundoy, bosque paramero en el filo de la Cordillera, La Cabaña, 2800 m, 2 Jan 1941, J.Cuatrecasas 11632 (holotype: F!; isotypes: COL!, P!, US!). IPNI ID: urn:lsid:ipni.org:names:77124997-1

***Blakea longisepala* (Gleason) Penneys & Judd, comb. nov.** Basionym: *Topobea longisepala* Gleason, Bull. Torrey Bot. Club 72: 392. 1945. **TYPE. COLOMBIA. Valle del Cauca:** Barco, Pacific coast, 5–80 m, 21–30 Apr 1944, J.Cuatrecasas 16975 (holotype: NY!; isotypes: F!, COL!). IPNI ID: urn:lsid:ipni.org:names:77124998-1

***Blakea macbrydei* (Wurdack) Penneys & Judd, comb. nov.** Basionym: *Topobea macbrydei* Wurdack, Phytologia 43: 354. 1979. **TYPE. ECUADOR. Morona-Santiago:** in cloud forest about one hour by trail from base camp at headwaters of Río Piuntza overlooking Río Zamora, NW range of Cordillera del Cóndor, 1850 m, 5 Jan 1972, B.MacBryde 963 (holotype: US!; isotypes: NY!, QCA!). IPNI ID: urn:lsid:ipni.org:names:77124999-1

***Blakea maguirei* (Wurdack) Penneys & Judd, comb. nov.** Basionym: *Topobea maguirei* Wurdack, Mem. N. Y. Bot. Gard 16: 43. 1967. **TYPE. ECUADOR. Guayas:** 54 miles east of Guayaquil, 540 m, 23 Sep 1959, B.Maguire & C.Maguire 44262 (holotype: US!; isotypes: COL!, NY!, US!). IPNI ID: urn:lsid:ipni.org:names:77125000-1

***Blakea maurofernandeziana* (Cogn.) Penneys & Almeda, comb. nov.** Basionym: *Topobea maurofernandeziana* Cogn., Monogr. Phan 7: 1193. 1891. **TYPE. COSTA RICA. Cartago:** Forêts de Juan Viñas, 25 Jan 1890, A.Tonduz 1844 (holotype: BR!; isotypes: CR, G!). IPNI ID: urn:lsid:ipni.org:names:77125001-1

***Blakea mcphersonii* (Almeda) Penneys & Almeda, comb. nov.** Basionym: *Topobea mcphersonii* Almeda, Brittonia 53: 163. 2001. **TYPE. PANAMA. San Blas:** San Blas boundary trail on Llano-Cartí road, ca. 350 m, 09°15'N, 79°00'W, 27 Jan 1986, G.McPherson & M.Merello 8176 (holotype: CAS!; isotypes: BM!, CR, EAP!, MEXU, MO!, PMA!, US!). IPNI ID: urn:lsid:ipni.org:names:77125002-1

***Blakea modica* (Wurdack) Penneys & Judd, comb. nov.** Basionym: *Topobea modica* Wurdack, Phytologia 48: 251. 1981. **TYPE. ECUADOR. Carchi:** El Pailon, ca. 45 km below Maldonado along a foot path to Tobar Donoso, 800 m, 28 Nov 1979, M.Madison & L.Besse 7095 (holotype: SEL!; isotypes: QCA!, US!). IPNI ID: urn:lsid:ipni.org:names:77125003-1

***Blakea mortoniana* (Wurdack) Penneys & Judd, comb. nov.** Basionym: *Topobea mortoniana* Wurdack, Phytologia 21: 129. 1971. **TYPE. COLOMBIA. Nariño:** wet cloud forest 7 km north of Altaquer along road to Barbacoas, 1250 m, 17 Oct 1969, B.Maguire & C.Maguire 61846 (holotype: NY (2 sheets)!; isotypes: COL!, K!, US!). IPNI ID: urn:lsid:ipni.org:names:77125004-1

***Blakea muricata* (Lozano) Penneys & Judd, comb. nov.** Basionym: *Topobea muricata* Lozano, Rev. Acad. Colomb. Cienc. Exact. 88: 342. 1999. **TYPE. COLOMBIA. Cauca:** Parque Nacional Natural Munchique, El Tambo, corregimiento La Romelia, camino al Observatorio, 2000 m, 1 Feb 1995, G.Lozano-C. et al. 6800 (holotype: COL!). IPNI ID: urn:lsid:ipni.org:names:77125005-1

***Blakea pascoensis* (Wurdack) Penneys & Judd, comb. nov.** Basionym: *Topobea pascoensis* Wurdack, Brittonia 40: 14. 1988. **TYPE. PERU. Pasco:** shrubby vegetation on ridge, trail to Chuchurras-Palcazu, headwaters of Río Tunqui, Prov. Oxapampa, 1900 m, 10°14'S, 75°28'W, R.Foster et al. 7745 (holotype: US!; isotypes: F!, MO!, NY!, TEX!). IPNI ID: urn:lsid:ipni.org:names:77125006-1

***Blakea pluvialis* (Standl.) Penneys & Almeda, comb. nov.** Basionym: *Topobea pluvialis* Standl., Publ. Field Mus. Nat. Hist., Bot. Ser. 22: 162. 1940. **TYPE. PANAMA. Darién:** Rain forest, crest of Cana-Cuasi trail, Chepigana District, 1200 m, 15 Mar 1940, M.Terry & R.Terry 1560 (holotype: F!; isotypes: BKL!, MO!). IPNI ID: urn:lsid:ipni.org:names:77125007-1

***Blakea sessilifolia* (Triana) Penneys & Judd, comb. nov.** Basionym: *Topobea sessilifolia* Triana, Trans. Linn. Soc. 28: 150. 1871 [1872]. **TYPE. COLOMBIA. Chocó:** J.Triana s.n. (holotype: BM!). IPNI ID: urn:lsid:ipni.org:names:77125008-1

***Blakea setosa* (Triana) Penneys & Judd, comb. nov.** Basionym: *Topobea setosa* Triana, Trans. Linn. Soc. 28: 149. 1871 [1872]. **TYPE. COLOMBIA. Nariño:** El Paramo inter Tuquerres et Barbacoas, 1100 m, May 1853, J.Triana 4093 (holotype: BM!; isotypes: COL!, K!, P!). IPNI ID: urn:lsid:ipni.org:names:77125009-1

***Blakea standleyi* (L.O.Williams) Penneys & Almeda, comb. nov.** Basionym: *Topobea standleyi* L.O. Williams, Fieldiana: Bot 29: 583. 1963. **TYPE. GUATEMALA. Baja Verapaz:** dry rocky hills in forest of pine and oak, north of Santa Rosa, 30 Mar 1939, P.Standley 69709 (holotype: F!; isotype: NY!). IPNI ID: urn:lsid:ipni.org:names:77125010-1

***Blakea stephanochaeta* (Naudin) Penneys & Judd, comb. nov.** Basionym: *Topobea stephanochaeta* Naudin, Ann. Sci. Nat. Bot. 3: 148. 1852. **TYPE. COLOMBIA.** Portochuelo, 1844, J.Goudot s.n. (holotype: P!; isotype: BR!). IPNI ID: urn:lsid:ipni.org:names:77125011-1

***Blakea steyermarkii* (Wurdack) Penneys & Judd, comb. nov.** Basionym: *Topobea steyermarkii* Wurdack, Act. Bot. Venez 1: 56. 1966. **TYPE. VENEZUELA. Sucre:** cloud forest on top of Cerro Patao, north of Puerto de Hierro and northeast of Güiria, Península de Paria, 1020 m, 19 Jul 1962, J.Steyermark & G.Agostini 91084 (holotype: US!; isotypes: F!, NY!, VEN!). IPNI ID: urn:lsid:ipni.org:names:77125012-1

***Blakea suaveolens* (Almeda) Penneys & Almeda, comb. nov.** Basionym: *Topobea suaveolens* Almeda, Proc. Calif. Acad. Sci 46: 323. 1990. **TYPE. PANAMA. Veraguas:** along trail to summit of Cerro Tute about 1/2 mile above the Escuela Agricultura Alto Piedra near Santa Fé , 900–1100 m, 29 Jan 1989, F.Almeda et al. 6484 (holotype: CAS!; isotypes: AAU, BM!, BR!, CR, DUKE, F!, MEXU, MICH, MO!, NY!, PMA!, TEX!, US!). IPNI ID: urn:lsid:ipni.org:names:77125013-1

***Blakea subbarbata* (Wurdack) Penneys & Judd, comb. nov.** Basionym: *Topobea subbarbata* Wurdack, Phytologia 6: 9. 1957. **TYPE. COLOMBIA. Valle del Cauca:** monte La Guarida, filo de la cordillera sobre La Carbonera (entre Las Brisas y Albán), Cordillera Occidental, vertiente occidental, 1950–2000 m, 17 Oct 1946, J.Cuatrecasas 22197 (holotype: NY!; isotypes: BC!, F!, P!, U!, US!). IPNI ID: urn:lsid:ipni.org:names:77125014-1

***Blakea subscabrula* (Triana) Penneys & Judd, comb. nov.** Basionym: *Topobea subscabrula* Triana, Trans. Linn. Soc. 28: 150. 1871 [1872]. **TYPE. COLOMBIA. Nariño:** in sylvis umbrosis inter Tuquerres et Barbacoas, 275–830 m, May 1853, J.Triana 4084 (holotype: BM!; isotypes: COL!). IPNI ID: urn:lsid:ipni.org:names:77125015-1

***Blakea subsessiliflora* (Wurdack) Penneys & Judd, comb. nov.** Basionym: *Topobea subsessiliflora* Wurdack, Phytologia 6: 10. 1957. **TYPE. COLOMBIA. Valle del Cauca:** Costa del Pacífico, río Cajambre, Barco, 5–80 m, 21–30 April 1944, J.Cuatrecasas 17191(holotype: NY!; isotypes: CAS!, F!, NY!, US!). IPNI ID: urn:lsid:ipni.org:names:77125016-1

***Blakea superba* (Naudin) Penneys & Judd, comb. nov.** Basionym: *Topobea superba* Naudin, Ann. Sci. Nat. Bot. 3: 147. 1852. **TYPE. COLOMBIA.** Combayma, 1844, J.Goudot s.n.(holotype: P!). IPNI ID: urn:lsid:ipni.org:names:77125017-1

***Blakea tetramera* (Almeda) Penneys & Almeda, comb. nov.** Basionym: *Topobea tetramera* Almeda, Proc. Calif. Acad. Sci., ser. 4, 52: 543. 2001. **TYPE. PANAMA. Veraguas:** headwaters of Río Caloveborita ca. 15 km past Escuela Agrícola Alto Piedra above Santa Fé, on the Atlantic watershed, 500 m, 16 May 1981, K.Sytsma & L.Anderson 4758 (holotype: CAS!; isotypes: MO!, PMA!). IPNI ID: urn:lsid:ipni.org:names:77125018-1

***Blakea tetroici* (Wurdack) Penneys & Judd, comb. nov.** Basionym: *Topobea tetroici* Wurdack, Phytologia 6: 10. 1957. **TYPE. COLOMBIA. Valle del Cauca:** Cordillera Occidental, vertiente occidental, hoya del río Dígua, Rio San Juan, abajo de Queremal a la derecha del río entre km. 52 y 53, 1300–1500 m, 19, 24, 27 Mar 1947, J.Cuatrecasas 23877 (holotype: F!; isotypes: P!, US!). IPNI ID: urn:lsid:ipni.org:names:77125019-1

***Blakea toachiensis* (Wurdack) Penneys & Judd, comb. nov.** Basionym: *Topobea toachiensis* Wurdack, Phytologia 38: 306. 1978. **TYPE. ECUADOR. Pichincha:** in virgin forest along Río Toachi near Santo Domingo, 700 m, 18 Jul 1963, C.Játiva & C.Epling 536 (holotype: US!; isotypes: CAS!, JEPS!, NY!, S, UC!). IPNI ID: urn:lsid:ipni.org:names:77125020-1

***Blakea trianae* (Cogn.) Penneys & Judd, comb. nov.** Basionym: *Topobea trianae* Cogn., Monogr. Phan 7: 1083. 1891. **TYPE. COLOMBIA. Nariño:** Barbacoas, 200 m, Apr 1853, J.Triana 4099 (holotype: G-DC!; isotypes: BM!, BR!, COL!, E!, K!, NY!, P!). IPNI ID: urn:lsid:ipni.org:names:77125021-1

***Blakea verrucosa* (Wurdack) Penneys & Judd, comb. nov.** Basionym: *Topobea verrucosa* Wurdack, Phytologia 38: 303. 1978. **TYPE. ECUADOR. Morona-Santiago:** Cordillera de Cutucú, western slopes, along a trail from Legroño to Yaupi, 2000 m, 02°46'S, 78°06'W, Nov 1976, M.Madison et al. 3566 (holotype: US!; isotype: US!). IPNI ID: urn:lsid:ipni.org:names:77125022-1

***Blakea watsonii* (Cogn.) Penneys & Almeda, comb. nov.** Basionym: *Topobea watsonii* Cogn., Monogr. Phan. 7: 1089. 1891. **TYPE. GUATEMALA.** **Izabal:** Hills on Chocon River, 11 Mar 1885, S.Watson 94/211 (holotype: BR). IPNI ID: urn:lsid:ipni.org:names:77125023-1

**Probable synonyms not transferred:**

*Topobea cuspidata* Gleason, *Topobea floribunda* Gleason, *Topobea pubescens* Gleason, *Topobea rhodantha* L.Uribe, *Topobea rupicola* Hoehne are probably synonyms of *Blakea parasitica* (Aubl.) D. Don, and so as to not contribute nomenclatural clutter, new combinations will not be made until their status has been confirmed. Likewise, *Topobea reducta* Gleason is likely a synonym of *Blakea alternifolia* (Gleason) Gleason and will not be transferred until further study.

**Ambiguous name:**

*Topobea andreana* Cogn. is not transferred at this time. This name is occupied by *Blakea andreana* Cogn. The species is poorly known, but was compared to *Topobea subscabrula* Triana by Cogniaux (1887), and to *Topobea grandiflora* Wurdack by Wurdack (1957). Study of the type is needed before a new combination can be proposed.

## Supplementary Material

XML Treatment for Blakeeae

XML Treatment for Chalybea

XML Treatment for Blakea
